# Pathological and Molecular Characterization of Grass Carp Co-Infected with Two *Aeromonas* Species

**DOI:** 10.3390/ani15020263

**Published:** 2025-01-18

**Authors:** Wenyao Lv, Zhijie Zhou, Lingli Xie, Xinyue Wang, Yifei Zhou, Lang Gui, Xiaoyan Xu, Yubang Shen, Jiale Li, Junqiang Qiu

**Affiliations:** 1 Key Laboratory of Freshwater Aquatic Genetic Resources, Ministry of Agriculture and Rural Affairs, Shanghai Ocean University, Shanghai 201306, China; lvwenyao2001@163.com (W.L.); 13816227337@163.com (Z.Z.); xllqff@163.com (L.X.); 17860285367@163.com (X.W.); yifeizhou97@gmail.com (Y.Z.); lgui@shou.edu.cn (L.G.); xyxu@shou.edu.cn (X.X.); ybshen@shou.edu.cn (Y.S.); jlli@shou.edu.cn (J.L.); 2 National Demonstration Center for Experimental Fisheries Science Education, Shanghai Ocean University, Shanghai 201306, China; 3 Shanghai Engineering Research Center of Aquaculture, Shanghai Ocean University, Shanghai 201306, China

**Keywords:** grass carp, co-infection, *Aeromonas*, metabolism, immune

## Abstract

The grass carp (*Ctenopharyngodon idella*) is highly susceptible to infections caused by *Aeromonas* species, particularly *A. hydrophila* and *A. veronii*. However, the immunological mechanisms underlying co-infection by these pathogens remain largely uncharted. This study investigated the pathogenesis and host immune response in grass carp following concurrent infection with *A. hydrophila* and *A. veronii*. Mortality was observed as early as 24 h post-infection, with cumulative mortality reaching 68%. Quantitative analysis demonstrated significantly elevated bacterial loads in hepatic tissue at 3 days post-infection (dpi). Histopathological evaluation revealed severe hepatic lesions characterized by cellular necrosis, cytoplasmic vacuolization, and hemorrhagic manifestations. Comparative transcriptomic analysis of hepatic tissues between co-infected and control specimens identified 868 and 411 differentially expressed genes (DEGs) at 1 and 5 dpi, respectively. Gene ontology and KEGG pathway analyses revealed significant enrichment of immune-related genes primarily associated with Toll-like receptor signaling and TNF signaling cascades. Notably, metabolic pathways showed substantial suppression while immune responses were significantly activated after infected. These findings provide novel insights into the host–pathogen interactions during *Aeromonas* co-infection in grass carp, which may facilitate the development of effective prevention and control strategies.

## Simple Summary

*Aeromonas hydrophila* and *Aeromonas veronii* are common pathogenic bacteria in grass carp aquaculture. Through comprehensive histopathological analysis and molecular characterization, we found that both pathogens caused severe cellular necrosis, cytoplasmic vacuolization, and hemorrhage in the liver of grass carp, similar to the manifestations of infection in other aquatic animals. The grass carp showed complex regulation of immune and metabolic responses during infection, especially activation of Toll-like receptors and TNF signaling pathways. Our findings provide new insights into host–pathogen interactions during *Aeromonas* co-infection in grass carp and provide a scientific basis for the development of prevention and control strategies.

## 1. Introduction

Grass carp (*Ctenopharyngodon idella*) represents one of the most economically valuable freshwater fish species in global aquaculture, with annual production exceeding 5.9 million tons [1]. However, bacterial infections pose substantial threats to grass carp farming, leading to significant economic losses in the aquaculture industry [2]. Among these pathogens, *Aeromonas hydrophila* and *Aeromonas veronii*, two representative species of the genus *Aeromonas* [3], are particularly concerning due to their ubiquitous presence in aquatic environments [4] and their ability to cause severe disease outbreaks. These opportunistic pathogens can induce hemorrhagic septicemia, skin ulceration, and internal organ damage in infected fish [5,6,7], significantly impacting aquaculture productivity.

Bacterial co-infections—defined as the simultaneous infection of a single host by multiple pathogens—have emerged as a significant concern in aquaculture. Recent studies have demonstrated that such co-infections frequently result in enhanced virulence and synergistic effects, leading to increased disease severity and higher mortality rates compared to single infections [8]. This phenomenon has been documented across various fish species. For instance, in zebrafish (*Danio rerio*), co-infection with *A. hydrophila* and *A. veronii* caused severe pathological changes, including renal tubular necrosis, tubular atrophy, and skin lesions, leading to higher mortality. The mortality rate in co-infected zebrafish was 87%, surpassing that of *A. hydrophila* (72%) or *A. veronii* (67%) alone [9]. Similar synergistic effects have been observed in other species: barramundi (*Lates calcarifer*) co-infected with *Streptococcus iniae* and *Shewanella algae* developed cutaneous ulcers and systemic disease [10]; koi carp (*Cyprinus carpio* var. *koi*) challenged with *Vibrio cholerae* and *A. veronii* exhibited multiple organ lesions and intestinal hemorrhage. However, koi subjected to infection with only *V. cholerae* showed only bleeding in the intestinal wall [11]; cobia (*Rachycentron canadum*) co-infected with *Photobacterium damselae* and *Vibrio harveyi* exhibited a 100% mortality rate, which was higher than the mortality rates observed with *Photobacterium damselae* (50%) or *Vibrio harveyi* (60%) alone [12]; and rainbow trout (*Oncorhynchus mykiss*) with concurrent *Pseudomonas fluorescens* and *Yersinia ruckeri* infections experienced an 80% mortality rate, which was higher than the mortality rates observed with *Pseudomonas fluorescens* (40%) or *Yersinia ruckeri* (60%) alone [13]. These studies underscore the significant impact of bacterial co-infections in fish and highlight the need for further research into their mechanisms and management strategies. These studies underscore the significant impact of bacterial co-infections in fish, demonstrating that such infections frequently result in enhanced virulence, synergistic pathogenic effects, and consequently, increased mortality rates with diverse clinical manifestations. Understanding the pathological changes and immune responses during such co-infections is crucial for developing effective preventive strategies in grass carp aquaculture.

Despite extensive research on bacterial co-infections in various fish species, the molecular mechanisms underlying host responses to such infections remain poorly understood, particularly in grass carp. There are numerous studies on single infections of *A. hydrophila* or *A. veronii* in grass carp [14,15,16,17]. However, to the best of our knowledge, there are no studies addressing co-infection by these two pathogens. Therefore, we believe that investigating the co-infection of *A. hydrophila* and *A. veronii* in grass carp is a novel and significant aspect of our research. To address this knowledge gap, we investigated the pathological changes and immune responses in grass carp liver during co-infection with *A. hydrophila* and *A. veronii*. Through comprehensive histopathological analysis and molecular characterization, this study aims to elucidate the key immune mechanisms activated during bacterial co-infection, thereby providing valuable insights for developing effective control strategies against these opportunistic pathogens in aquaculture.

## 2. Materials and Methods

### 2.1. Fish and Challenge Experiments

Healthy grass carp (*Ctenopharyngodon idella*) were from Suzhou Shenhang Eco-technology Development Limited Company (Wujiang District, Suzhou, China). A total of 60 healthy grass carp, each weighing 70–80 g, were selected. The fish were acclimated in 50 cm × 40 cm × 35 cm glass aquaria under controlled laboratory conditions for one week before the experiments commenced. *Aeromonas hydrophila* (23091906bs) and *Aeromonas veronii* (23090701bs) strains were sourced from the National Pathogen Collection Center for Aquatic Animals, Shanghai Ocean University. Both bacterial strains were cultured in a Luria–Bertani (LB) medium at 28 °C for 24 h with constant shaking. Bacterial cells were harvested via centrifugation, washed with sterile phosphate-buffered saline (PBS), and resuspended in an LB medium to achieve a final concentration of 1 × 10^7^ CFU/mL.

For challenge experiments, a total of 60 healthy grass carp were randomly divided into two groups (co-infection and control) with 3 replicates (10 fish per replicate, *n* = 30 per group). Fish in the co-infection group were intraperitoneally (i.p.) injected with 200 μL of a bacterial suspension mixed in the same ratio (1 × 10^7^ CFU/mL), while control fish received an equal volume of sterile PBS. Liver were collected at 1, 3, 5, and 7 days post-infection (dpi). At each time point, three randomly selected fish from each group were euthanized with MS-222, and their livers were immediately fixed in 4% paraformaldehyde for histopathological examination. The remaining liver samples were flash-frozen in liquid nitrogen, and stored at −80 °C until further processing.

### 2.2. Histological and Bacterial Load Determination

For the histopathological examination, the liver was fixed in 4% paraformaldehyde. Briefly, tissues were dehydrated through a graded ethanol series, embedded in paraffin wax, sectioned at 5 μm thickness, and stained with hematoxylin and eosin (H&E) [18]. Histological changes were examined and photographed using a light microscope.

The bacterial load in the liver was determined using the plate counting method. Briefly, liver samples were weighed and homogenized in sterile saline. Serial dilutions of the homogenates were prepared, and 50 μL of each dilution was plated onto LB agar plates supplemented with ampicillin. Plates were incubated at 28 °C for 24 h, after which colony-forming units (CFU) were counted.

### 2.3. Transcriptome Analysis

Total RNA was extracted from liver samples using TRIzol reagent (Invitrogen, Carlsbad, CA, USA) following the manufacturer’s protocol. The RNA quality was assessed using three methods: purity was evaluated using a NanoDrop 2000 spectrophotometer (Thermo Fisher Scientific, Waltham, MA, USA), concentration was measured using a Qubit fluorometer (Invitrogen, Carlsbad, CA, USA), and integrity was assessed using an Agilent 2100 Bioanalyzer (Agilent Technologies, Inc., Santa Clara, CA, USA). RNA integrity number(s) (RIN) >8.0 were used for library construction. RNA libraries were prepared using the VAHTS Universal V6 RNA-seq Library Prep Kit following the manufacturer’s instructions.

Libraries were sequenced on an Illumina NovaSeq 6000 platform to generate 150 bp paired-end reads. Raw sequencing data were filtered using FastP [19] to remove low-quality reads, adaptor sequences, and contaminating sequences. Clean reads were aligned to the grass carp reference genome using Hisat2 [20]. Differential expression analysis was performed using the DESeq2 [21]. Genes with a fold change ≥2 and adjusted *p*-value < 0.05 were considered differentially expressed. Functional enrichment analysis of differentially expressed genes (DEGs) was conducted using the ClusterProfiler [22], focusing on the Kyoto Encyclopedia of Genes and Genomes (KEGG) pathways. Protein–protein interaction (PPI) networks were drawn using STRING 12.0 (https://cn.string-db.org/, accessed on 3 September 2024) and Cytoscape (v3.10.2) [23].

### 2.4. Statistical Analysis

Statistical analysis was performed using GraphPad Prism 9 (GraphPad Software Inc., USA) [24]. The experimental data’s mean and standard deviation (mean ± SD) are expressed. The results of hepatic bacterial load were statistically analyzed by employing one-way ANOVA and Tukey multiple comparison post hoc test. The tests showed * *p* < 0.05, ** *p* < 0.01, and *** *p* < 0.001 for significant differences.

## 3. Results

### 3.1. Survival Rates and Bacterial Load

**Figure 1:**
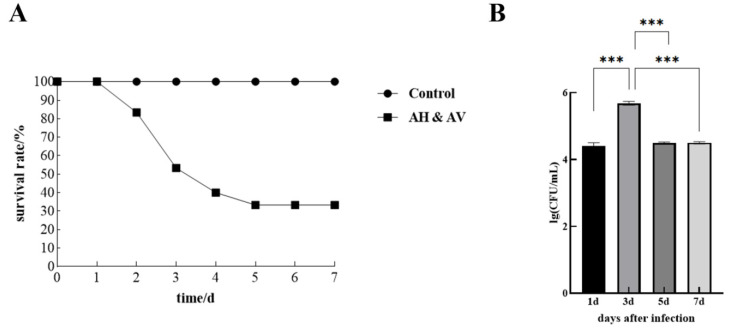
Survival curve and hepatic bacterial load changes in grass carp co-infected with *A. hydrophila* and *A. veronii*. (**A**) Survival curve of grass carp co-infected with *A. hydrophila* and *A. veronii*. (**B**) Changes in hepatic bacterial load in grass carp co-infected with *A. hydrophila* and *A. veronii.*(“***” indicates *p* < 0.001).

To investigate the pathogenicity of Aeromonas co-infection, healthy grass carp were experimentally challenged with a mixed bacterial suspension containing *A. hydrophila* and *A. veronii*. Initial mortality was observed at 1-day post-infection (dpi), followed by a sharp increase in mortality rate that peaked at 3 dpi. The mortality pattern plateaued after 5 dpi, yielding a final survival rate of 32%. Throughout the experimental period, no deaths were recorded in the control group (Figure 1A). To determine the bacterial colonization dynamics, we quantified the bacterial load in the liver at four time points post-infection. The bacterial load exhibited a time-dependent pattern, reaching its apex at 3 dpi (4.8 × 10^5^ CFU). Comparatively lower bacterial loads were detected at 1, 5, and 7 dpi, measuring 2.6 × 10^4^, 3.2 × 10^4^, and 3.1 × 10^4^ CFU, respectively (Figure 1B). Statistical analysis was performed using one-way ANOVA followed by Tukey’s post hoc test to determine significant differences between time points (Figure 1B). The results indicate that the bacterial load was significantly higher at 3 dpi compared to the other time points. Specifically, bacterial loads at 1 dpi, 5 dpi, and 7 dpi were significantly lower than at 3 dpi (*p* < 0.05), highlighting the peak bacterial colonization at 3 dpi.

### 3.2. Histomorphology

**Figure 2:**
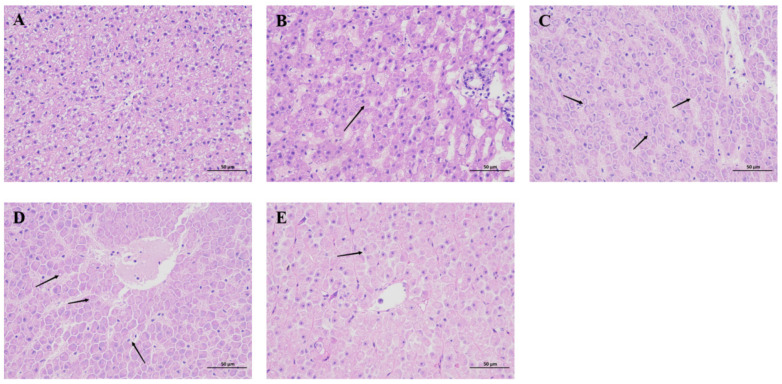
Histological effects of co-infection of *A. hydrophila* and *A. veronii* on liver of grass carp. (**A**–**E**): infected for 0, 1, 3, 5, and 7 days. The arrows indicate apoptotic cells.

In the control group, hepatocyte structure and arrangement were normal (Figure 2A). At 1 dpi, minor bleeding was observed in the liver tissue (Figure 2B). At 3 dpi, a large number of hepatocytes were observed to be necrotic, and a large number of nuclei disappeared in the livers of grass carp. During this period, some of the liver cell walls disappeared, and the shape of hepatocytes became irregular (Figure 2C). At 5 dpi, necrosis and cavitation gradually subsided, and new cells were regenerated (Figure 2D). At 7 dpi, necrosis and cavitation had resolved, and large numbers of new cells grew. In addition, the connection between blood vessels and hepatocytes was intact (Figure 2E).

### 3.3. Transcriptome

**Figure 3:**
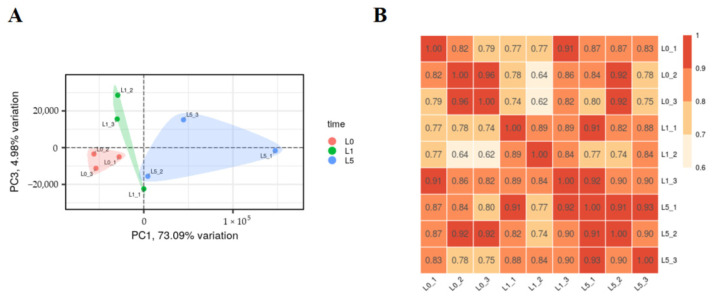
Sample relationship analysis. (**A**) Principal component analysis (PCA) of the genes in terms of variance across samples. (**B**) Sample correlation heat map.

High-throughput sequencing generated approximately 412 million clean reads. The mapping rates consistently exceeded 95.90%, Q30 percentages ranged from 93.98% to 94.92%, and GC content ranged from 45.51% to 46.68% (Table 1). Both principal component analysis (PCA) (Figure 3A) and Pearson’s correlation analysis (Figure 3B) revealed distinct clustering patterns between control and infected groups, with high reproducibility among biological replicates.

### 3.4. DEGs and Enrichment Analysis

**Figure 4:**
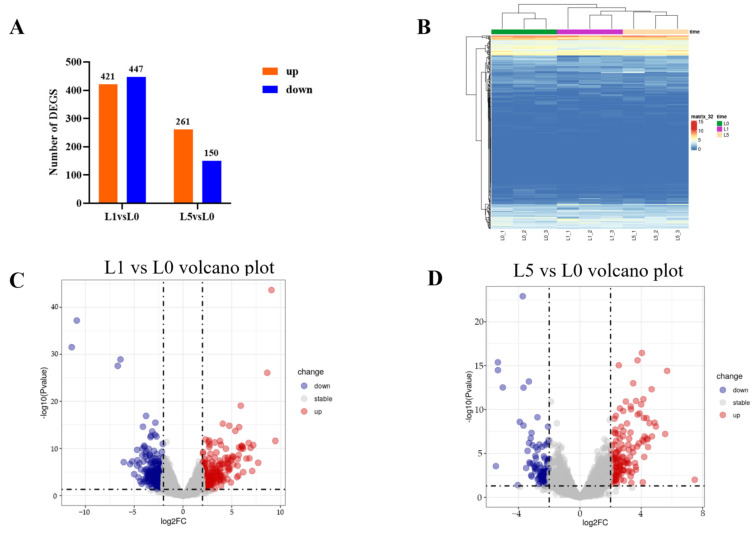
DEGs expression analysis. (**A**) Summary of differential gene expression between two experimental groups. (**B**) Expression pattern clustering analysis of differentially expressed genes. (**C**) Volcano plot of DEGs in the L1 vs. L0 group. (**D**) Volcano plot of DEGs in the L5 vs. L0 group.

**Figure 5:**
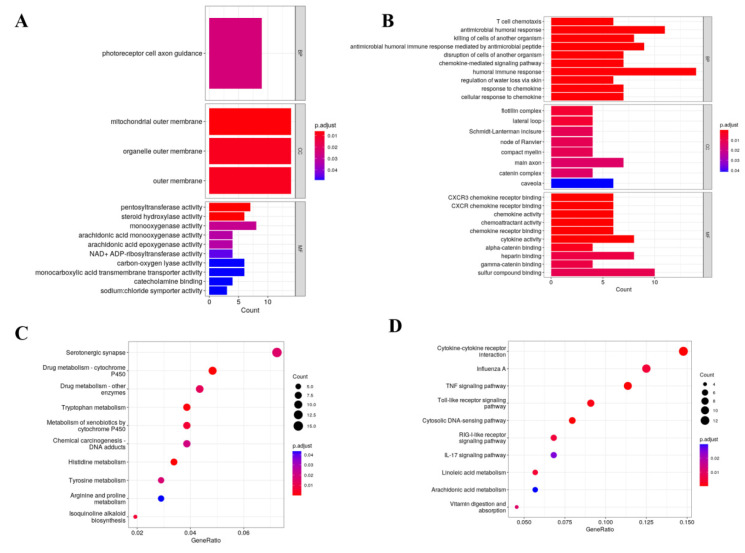
GO and KEGG function enrichment analysis of DEGs. (**A**) GO function enrichment analysis of the L1 vs. L0 group (top 10 enriched terms). (**B**) GO function enrichment analysis of the L5 vs. L0 group (top 10 enriched terms). (**C**) KEGG function enrichment analysis of the L1 vs. L0 group (top 10 enriched terms). (**D**) KEGG function enrichment analysis of the L5 vs. L0 group (top 10 enriched terms).

Differential expression analysis identified 868 DEGs at 1 dpi (421 up-regulated and 447 down-regulated) and 411 DEGs at 5 dpi (261 up-regulated and 150 down-regulated) (Figure 4A), as illustrated by volcano plots (Figure 4C,D). Hierarchical clustering analysis of these DEGs demonstrated distinct expression patterns between infected and control groups (Figure 4B). GO enrichment analysis at 1 dpi revealed significant enrichment in membrane-associated components and metabolic functions, including pentosyltransferase and steroid hydroxylase activities (Figure 5A). At 5 dpi, enriched terms were predominantly immune-related, including chemokine activity and T cell immune response (Figure 5B). Similarly, KEGG pathway analysis showed enrichment in metabolic pathways at 1 dpi, particularly in histidine and tryptophan metabolism (Figure 5C), while immune-related pathways, including toll-like receptor and TNF signaling, were significantly enriched at 5 dpi (Figure 5D).

### 3.5. Protein–Protein Interaction Networks

**Figure 6:**
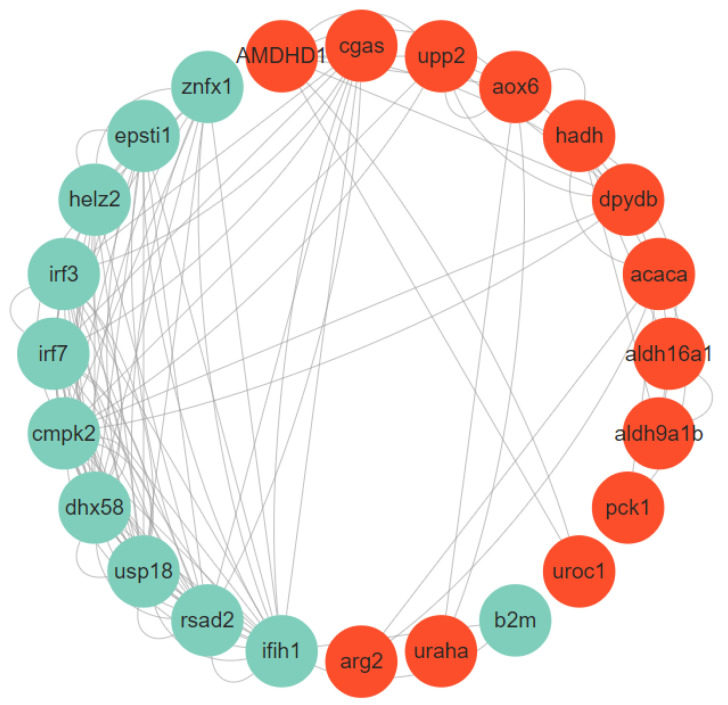
PPI networks of selected key DEGs. The red color indicates the metabolism-related DEGs; the green color indicates the immune-related DEGs.

KEGG pathway enrichment analysis revealed significant dysregulation of metabolic and immune-related genes during infection. Notably, metabolic genes (*amdhd1*, *aox1*, *urah*, *aldh16a1*, and *hadh*) showed substantial suppression, while immune-related genes (*irf3*, *irf7*, *epsti1*, *znfx1*, *cmpk2*, *cxcl11*, and *rsad2*), were significantly activated in infected specimens (Table 2). Protein–protein interaction analysis demonstrated extensive crosstalk between these dysregulated metabolic and immune pathways during bacterial infection (Figure 6).

## 4. Discussion

Bacterial co-infection represents a significant challenge in aquaculture, frequently leading to complex disease manifestations and altered pathogenesis [25]. Although such co-infections substantially impact fish health, their pathogenic mechanisms and host–pathogen interactions remain poorly understood. Co-infection, defined as the simultaneous infection of a host by multiple genetically distinct pathogens [26,27], can result in either antagonistic or synergistic interactions between pathogens [26], with the latter typically causing more severe clinical outcomes. These outcomes are characterized by enhanced disease severity, elevated mortality rates, modified host susceptibility patterns, and extended infection periods [28]. In the present study, we investigated the co-infection of grass carp with *Aeromonas hydrophila* and *Aeromonas veronii*, focusing on the liver as a primary site of bacterial colonization and immune response [29,30]. Our results demonstrated that bacterial loads peaked at three days post-infection (dpi), correlating with maximum mortality rates that ultimately reached 68%, consistent with previous observations by Sarkar et al. [31]. The enhanced pathogenicity of bacterial co-infections has been documented across various fish species. For instance, Chandrarathna et al. [9] reported that co-infection of zebrafish with multidrug-resistant strains of *A. hydrophila* and *A. veronii* resulted in significantly higher mortality compared to single infections. Similarly, in striped catfish, co-infection with *Edwardsiella ictaluri* and *A. hydrophila* induced 95% cumulative mortality, substantially exceeding the mortality rates observed in single infections (80% for *E. ictaluri* and 10% for *A. hydrophila*). These comparative findings strongly suggest synergistic pathogenic effects during bacterial co-infections.

This study highlights the key adaptive immune response of grass carp in co-infection with *A. hydrophila* and *A. veronii*, revealing the underlying molecular mechanisms of host response and defense against invasion. Thus, pathogen interactions can cause changes in bacterial load. To elucidate the pathological consequences of bacterial co-infection, we conducted a comprehensive histopathological analysis of infected liver tissues. Previous studies have demonstrated that single infections with *Aeromonas hydrophila* induce hepatocyte swelling and vacuolation in grass carp [32], and cause vacuolar degeneration with nuclear consolidation and ferric hemoflavin accumulation in Nile tilapia [33]. In our study, co-infection with *A. hydrophila* and *A. veronii* resulted in progressive liver damage characterized by severe hepatocyte necrosis, extensive vacuolar degeneration, and nucleolysis. These pathological changes are attributed to a robust inflammatory response initiated by the liver, where infiltrating inflammatory cells release various cytokines and enzymes that contribute to hepatocyte damage. The compromised metabolic functions of hepatocytes subsequently lead to lipid accumulation and further vacuolar degeneration. Notably, while the observed hepatocellular necrosis, vacuolization, and hemorrhage were similar to those reported in single *A. hydrophila* infections [16], the extent and progression of tissue damage in co-infected fish were markedly more severe.

To elucidate the molecular mechanisms underlying host defense, we analyzed the transcriptional responses in grass carp during bacterial co-infection. Functional enrichment analysis revealed concurrent modulation of metabolic pathways—specifically histidine and tryptophan metabolism—and immune pathways, particularly the Toll-like receptor and TNF signaling cascades. The immune response was predominantly mediated through innate immunity [34], with significant activation of the Toll-IFN signaling pathway [35,36]. It has been shown that the flagellum of Aeromonas is one of its major virulence factors [37]. TLR5, a member of the Toll-like receptor family, recognizes flagellin produced by the bacteria, which activates the host immune system and induces a defense response against the bacteria [38,39]. Key components of the Toll-IFN pathway showed differential expression, including marked upregulation of *usp18* in infected livers, suggesting enhanced immune response to bacterial invasion. The pathway activation cascade involves *ifih1*-mediated transcription of *irf1*—one of three key interferon regulatory factors alongside *irf3* and *irf7* [40], leading to increased antimicrobial defense and inflammatory cytokine production [41,42,43]. Concurrent with immune pathway activation, we observed significant alterations in metabolic regulators. Notably, *amdhd1*, which mediates histidine and tryptophan metabolism [44] and liver development [45], showed marked downregulation, potentially compromising cellular metabolism and triggering stress-induced cell death. Similarly, reduced expression of *aox1*, a tryptophan metabolite marker with antioxidant properties [46], may impair cellular protection against oxidative stress. These metabolic changes are particularly relevant as tryptophan and histidine regulate T-cell proliferation [47] and cytokine production [48], respectively. A key finding was the significant upregulation of *cmpk2* following co-infection with *A. hydrophila* and *A. veronii*. Through NLRP3 inflammasome activation [43], elevated *cmpk2* expression correlated with reduced bacterial colonization in hepatocytes, as confirmed by histopathological and bacterial load analyses. These findings demonstrate the intricate coordination between immune and metabolic responses in countering bacterial co-infection.

## 5. Conclusions

In this study, we demonstrated that co-infection with *A. hydrophila* and *A. veronii* results in severe hepatic damage in grass carp, manifested by extensive hepatocyte necrosis, vacuolization, and hemorrhage. Gene ontology and KEGG pathway analyses revealed the synchronized modulation of immune and metabolic processes during co-infection, with significant enrichment of immune-related genes primarily associated with Toll-like receptor signaling and TNF signaling pathways, indicating a complex host response. These findings highlight the complex host immune response to bacterial co-infection, emphasizing the need for improved prophylactic strategies in grass carp aquaculture to mitigate infections by these opportunistic pathogens.

## Figures and Tables

**Table 1:** Transcriptome sequencing data evaluation and statistical analysis.

Sample	Clean Reads	Mapped Reads	Mapped (%)	GC Content (%)	Q30 (%)
L0_1	42,039,400	40,701,314	96.82	46.05	94.70
L0_2	42,851,546	41,490,832	96.82	46.68	94.92
L0_3	44,939,124	43,334,704	96.43	46.33	94.55
L1_1	45,719,910	43,887,889	95.99	45.51	94.28
L1_2	47,494,342	45,660,941	96.14	45.52	94.44
L1_3	46,304,186	44,664,653	96.46	46.03	94.58
L5_1	47,555,602	45,991,019	96.71	46.21	94.84
L5_2	48,151,050	46,446,187	96.46	46.05	94.79
L5_3	47,017,870	45,089,842	95.90	45.72	93.98

**Table 2:** The key candidate DEGs shared in two comparison groups.

ID	Description	Abbreviation	Log2 Fold Change	
			L1 vs. L0	L5 vs. L0
Cide__011375	Epithelial stromal interaction 1 (EPSTI1)	EPSTI1	4.00	3.31
Cide__020487	Zinc finger NFX1-type containing 1 (ZNFX1)	ZNFX1	3.78	3.30
Cide__020440	HELZ2 (Helicase with zinc finger 2)	HELZ2	3.04	2.91
Cide__001364	DHX58 (DEXH box helicase 58)	DHX58	2.12	2.30
Cide__023962	Ubiquitin specific peptidase 18 (USP18)	USP18	3.65	2.76
Cide__017820	Cytidine/uridine monophosphate kinase 2 (CMPK2)	CMPK2	4.52	3.89
Cide__029329	Interferon regulatory factor 3 (IRF3)	IRF3	3.09	3.00
Cide__031701	Interferon regulatory factor 7 (IRF7)	IRF7	4.62	4.35
Cide__017821	Radical S-adenosyl methionine domain containing 2 (RSAD2)	RSAD2	5.09	4.08
Cide__010428	Interferon induced with helicase C domain 1 (IFIH1)	IFIH1	2.87	2.20
Cide__023845	Beta-2 microglobulin (B2M)	B2M	2.69	2.34
Cide__002970	Aminoadipate-semialdehyde dehydrogenase 1 (AMDHD1)	AMDHD1	−3.57	−1.67
Cide__005141	Aldehyde oxidase 1 (AOX1)	AOX1	−2.23	−1.21
Cide__022550	Arginase 2 (ARG2)	ARG2	2.45	1.67
Cide__001177	Aldehyde dehydrogenase 16 family member A1 (ALDH16A1)	ALDH16A1	−2.21	−1.39
Cide__015820	Urate hydroxylase (URAH)	URAH	−2.94	−1.12
Cide__020438	Phosphoenolpyruvate carboxy kinase 1 (PCK1)	PCK1	2.49	5.36
Cide__009138	Hydroxy acyl-CoA dehydrogenase (HADH)	HADH	−2.17	−1.03
Cide__001608	Karyopherin alpha 2 (KPNA2)	KPN2A	2.27	2.41
Cide__010370	RAN binding protein 2 (RANBP2)	RANBP2	4.68	3.16
Cide__007370	C-X-C Motif Chemokine Ligand 11(CXCL11)	CXCL11	2.30	3.01
Cide__028555	Mitogen-Activated Protein Kinase Kinase 6(MAP2K6)	MAP2K6	−2.75	−2.56

## Data Availability

The original contributions presented in this study are included in the article. Further inquiries can be directed to the corresponding author.
